# Smoking Addiction in Patients with Schizophrenia Spectrum Disorders and Its Perception and Intervention in Healthcare Personnel Assigned to Psycho-Rehabilitation Programs: A Qualitative Research

**DOI:** 10.3390/healthcare10112275

**Published:** 2022-11-13

**Authors:** Pasquale Caponnetto, Marilena Maglia, Marta Mangione, Chiara Vergopia, Graziella Chiara Prezzavento, Riccardo Polosa, Maria Catena Quattropani, Jennifer DiPiazza, Maria Salvina Signorelli

**Affiliations:** 1 Department of Educational Sciences, Section of Psychology, University of Catania, 95121 Catania, Italy; 2 Center of Excellence for the Acceleration of Harm Reduction (COEHAR), University of Catania, 95121 Catania, Italy; 3 CTA-Villa Chiara Psychiatric Rehabilitation Clinic and Research, 95030 Mascalucia, Italy; 4 ECLAT Srl, Spin-Off of the University of Catania, 95121 Catania, Italy; 5 Department of Clinical and Experimental Medicine, University of Catania, 95121 Catania, Italy; 6 Hunter Bellevue School of Nursing, Hunter College, City University of New York, New York, NY 10065, USA

**Keywords:** smoking addiction, schizophrenia, healthcare personnel, qualitative research, e-cigarette

## Abstract

Patients with schizophrenia spectrum disorders have a higher prevalence and frequency of smoking rates when compared to the rest of the population; to this, it must be added that they develop a greater dependence and have some worse health consequences than the general population. This is qualitative research on the perception of smoking in healthcare professionals assigned to psycho-rehabilitation programs for patients with schizophrenia spectrum disorders. The point of view of health personnel (Psychologists, Psychiatrists, Pedagogists, and Nurses) about cigarette smoking in these patients was analyzed, focusing on their implications in disturbance and comparing them with e-cigarettes too. The methodology used to collect the data was a semi-structured interview with five questions. The research path was carried out in two assisted therapeutic communities that are clinics for the rehabilitation of serious mental illness in the period between November and July 2022. The results showed that the opinion of health professionals on smoking is very negative. Research has also shown that nearly all patients are smokers; however, their high grade of addiction is caused by periods of high stress due to various factors that lead patients to consume a greater number of cigarettes. Almost all respondents have a positive opinion of the e-cigarette, which was defined as an excellent substitute for traditional cigarettes.

## 1. Introduction

Schizophrenia is one of the top 20 causes of disability and is characterized by disorganized thinking, distorted perception, difficulty in focusing attention, lack of emotional expression, and behavioral alterations. Those who suffer from it can isolate themselves from others to create a world for themselves made up of strange beliefs (delusions) and hallucinations [1]. It usually starts in late adolescence/early adulthood, and people with schizophrenia show a certain number of acute episodes and, between episodes, less severe symptoms but which are still very debilitating. Symptoms experienced by schizophrenic patients can be positive, negative, and disorganized. Positive symptoms include delusions and hallucinations; negative symptoms refer to apathy, anhedonia, alogia, and affective flattening; disorganized symptoms refer to catatonic behaviour and disorganized speech. The DSM-5 requires at least 6 months of continuing symptoms for it to arrive at the diagnosis of the disorder [2]. Schizophrenia is also associated with increased mortality, with a shortened lifespan and standardized mortality ratios that are reported to be two-fold to four-fold those of the general population [3]. The common co-occurrence of other psychiatric disorders, including substance use disorders [4], contributes to morbidity and mortality among individuals with schizophrenia. Patients with schizophrenia spectrum disorders have a higher smoking prevalence and frequency when compared to the rest of the population; it must be added that this includes greater dependence and worse health consequences than the general population [5]. A meta-analysis of five studies in four countries determined that tobacco smoking was associated with a diagnosis of schizophrenia (OR = 5.9) with heavier smoking, greater nicotine dependence, and less cessation than controls of the general population [6]. Even patients with the first episode of psychosis are more likely to smoke than same-age controls, as confirmed in a meta-analysis [7]. As a result of high smoking rates, people with serious mental health conditions also have high mortality rates compared to the general population. Therefore, quitting smoking is especially important for this group of people since smoking is the biggest cause of these patients’ shortened life expectancy [8]. Smokers with schizophrenia die frequently, especially from associated smoking diseases (15–20 years earlier than the general population) [9]. Callaghan et al. [10] demonstrated in a large-scale follow-up study that conditions related to tobacco smoking comprised approximately 53% (23,620/44,469) of total deaths in the schizophrenic spectrum disorder sample, 48% (6004/12,564) in patients with bipolar disorder, and 50% (35,729/71,058) in people with depression. This included an increase in the risk of deaths related to cancer due to tobacco use (standardized mortality rate (SMR) 1.30, 95% CI 1.3–1.4), cardiovascular disease (SMR 2.46, 95% CI 2.41–2.50) and respiratory diseases (SMR 2.45, 95% CI 2.41–2.48).

Physicians play a primary role in patient smoking cessation. The national exploratory survey conducted in the US by Steinberg et al. regarding the knowledge and the communication on the use of tobacco by the physician to their patients shows how there are widespread misperceptions about nicotine, considered harmful and directly related to the development of tumours [11]. This overestimation of the harms caused by nicotine may be exacerbated by a potential availability bias that nicotine is the cigarette constituent most familiar to many people [12]. The study by Delnevo et al. investigated how health professionals think about the e-cigarette as a useful smoking cessation device for their patients. The finding suggests that doctors who approved a harm reduction perspective or who had ever smoked were more likely to recommend e-cigarettes to patients. In hypothetical clinical scenarios, doctors were more likely to recommend e-cigarettes for an older smoker with previous unsuccessful quit attempts and the use of pharmacotherapy for a younger, light smoker with no previous cessation treatments [13]. In contrast to the US, physicians in the UK are encouraged to consider e-cigarettes as an option for smoking cessation and to support smokers who wish to try them [14].

Recently, evidence has shown that smoking can have a detrimental effect on working memory [15] and hippocampal volume [16] among people with schizophrenia spectrum disorders. A recent systematic review and comparative meta-analysis of studies conducted by Wang et al. [17] explored cognitive functions in smokers and non-smokers with schizophrenia spectrum disorders and it was found that smokers with schizophrenia had lower neurocognitive performance in cognitive tasks compared to non-smokers with schizophrenia too. In a recent systematic review and meta-analysis, Huang et al. [18] explored the effect of traditional cigarette smoking on various psychopathological patients with negative symptoms of schizophrenia. Their meta-analysis of 24 studies looked at positive and negative symptoms with the positive and negative symptoms of schizophrenia scale (PANSS) or the positive symptom rating scale (SAPS) and the scale for negative symptom assessment (SANS) in smokers and non-smokers with schizophrenia spectrum disorders and showed that smokers had symptoms more severe positives than non-smokers but the same study found no significant difference between smokers and non-smokers with negative symptoms. In schizophrenia spectrum disorders, cigarette smoking is traditionally associated with more severe psychopathological symptoms and increased hospital admissions. Kobayashi et al. [19] conducted a retrospective study with 460 discharged patients with schizophrenia spectrum disorders in Japan. Smoking hospital psychiatric readmissions were reviewed and it was noted that hospital readmission rates were higher in smokers with schizophrenia compared with smokers without schizophrenia (HR = 1.78). The participants were freely admitted to psychiatric hospitals for the first time and the results cannot be generalized to other populations or people in different stages of this disease. Smoking is also associated with an increased need for drug doses. Smoking increases the activity of the cytochrome P450 1A2 (CYP1A2) hepatic enzyme system, thereby reducing blood concentrations of many drugs [20] and this process can also have an impact on antipsychotic drugs with a meta-analysis concluding that two antipsychotics, olanzapine, and clozapine, should be increased by 30% and 50%, respectively [21].

A substantial number of studies have presented a considerable number of reasons for high rates of smoking and high nicotine dependence in smokers with schizophrenia spectrum disorders. In this case, nicotine evokes its physiological effects by binding with acetylcholine receptors to nicotine (nAChRs) and boosts the rewards from brain stimulation [22]. The nAChR also plays an essential role in cognitive processes such as memory and learning and researchers demonstrated anomalies of the nAChR in people with schizophrenia [23]. Schizophrenia is linked to high levels of dopamine in the dorsal striatum and reduced cortical dopamine release [24]. Dopamine is a neurotransmitter system influenced by nAChRs. All antipsychotic drugs act on the dopaminergic system by blocking dopamine receptors of the D2-type family [25]. Nicotine increases dopamine levels in the striatum by stimulating its release through nicotinic receptors and decreasing its release degradation by inhibiting monoamine oxidase A and B. These produce an effect of stimulation and a reduction of antipsychotic extrapyramidal side effects [20]. It was suggested that in people with schizophrenia spectrum disorders, traditional cigarette smoking is a way to self-medicate by reducing problems associated with antipsychotic treatment and reducing positive and negative psychotic symptoms [26] by seeking to remedy cognitive performance as a result of the symptoms’ underlying disorders of the schizophrenic spectrum and stimulating attention and working memory [27]. Kumari and Postma [28] have suggested that the smoking rate in people with schizophrenia is increased due to the enhancing effect of nicotine on symptoms of schizophrenia. Studies described the positive neurocognitive effects of nicotine in the main cognitive domains (attention, processing speed, working memory, and ability psychomotor) in individuals with cognitive disorders with schizophrenia. Research conducted by Barr et al. [29] examined the effect of transdermal nicotine (14 mg nicotine patches) and placebo in non-smokers with schizophrenia (*n* = 28) and healthy controls (*n* = 32) in an internal study and showed that nicotine improved cognitive performance in both groups in terms of attention, but patients with schizophrenia showed greater improvement in inhibition and impulse control compared to healthy controls. In a second study, Jubelt et al. [30] studied the effect of transdermal nicotine on the performance of episodic memory in non-smoking individuals with (*n* = 10) and without schizophrenia (*n* = 12). Compared to the placebo control conditions, both groups increased in processing speed and accuracy in recognizing new objects but there was a trend for a stronger nicotine-induced effect in schizophrenic patients’ reduction of false alarms, which is important considering that memory deficits are associated with functional impairment in schizophrenia and that the detection of altered novelty was linked to positive symptoms of schizophrenia. However, it is important to consider that these results refer to non-smokers with schizophrenia using nicotine not released from traditional cigarettes. Further studies evaluated the impact of nicotine intake on function cognitive impairment in people with schizophrenia spectrum disorders [31]. Despite the differences in the level of nicotine addiction, the severity of the withdrawal of nicotine, craving, and satiety, and the method of nicotine administration (gum, transdermal patch, nicotine nasal spray) among participants in different research studies, the results suggest that nicotine administration has a role in improving cognition in schizophrenia, particularly for attention/alertness [32]. However, none of these studies used cognitive psychodiagnostic tools specifically designed for people suffering from schizophrenia spectrum disorders, such as the brief assessment of cognition in schizophrenia (BACS) [33], or MATRICS Consensus Cognitive Battery (MCCB) scale [34], but instead, they used psychodiagnostic tools created for the general population as the repeatable battery for the evaluation of the neuropsychological status (RBANS) [35], Spatial attentional Resource Allocation Task (SARAT) and Singleton Detection Task or Continuous Performance Task (CPT). A Spanish study explored the association between smoking frequency and the severity of positive symptoms with the number of hospitalizations among 250 outpatients with schizophrenia [36]. Patients were classified into three categories: self-employed, mildly dependent, and non-dependent smokers. High PANSS total scores and positive symptoms were less frequent in addicts than in non-smokers or dependent smokers. Addicted smokers had the worst results. Aguilar argued that their data did not support the self-medication hypothesis but suggested a complex interaction between nicotine addiction and symptoms of schizophrenia.

## 2. Materials and Methods

### Participants and Procedures

This study used a qualitative approach with which it was possible to investigate the point of view of the health personnel regarding cigarettes, their perception of the importance of these for patients, and the comparison with e-cigarettes. Qualitative research is a method of investigation based on observation or interaction with participants to understand the reasons for their behaviors. Information is collected through audio, video, and transcribed text from interviews with individual participants or focus groups. After collecting the information, we then moved on to the interpretation of the information and the presentation of the participants’ points of view. The sample was chosen based on eligibility criteria, which in this case were: being part of the medical team of an assisted therapeutic community (C.T.A.) a clinic for the rehabilitation of serious mental illness; being in daily contact with patients; observing patients using cigarettes; having observed patients while using e-cigarettes. All the interviews were carried out in the C.T.A. where the interviewees work. The interviews were carried out in the meeting rooms of the centers and the setting provided only the presence of the interviewee and the interviewer, with the task of investigating the various aspects that were reported as points of interest for qualitative research. The environments in which the interviews were carried out are presented as bright and welcoming rooms; they can accommodate long tables with chairs placed side by side or with chairs facing each other. The interviews involved 20 operators including psychiatrists, psychologists, pedagogists, social workers, and technicians working in the C.T.A. The sample was divided as shown in the following table (Table 1). The methodology used to collect information regarding the operators’ point of view was a semi-structured interview, which included five research questions (Table 2). These questions were starting points that helped the operators to reflect on some aspects concerning the pathology, such as, for example, the rituality and repetition of the gesture and the idea of the cigarette as a moment of sharing and community among the various patients; the latter has allowed us to better understand the value of the cigarette for psychiatric patients.

Individual structured interviews were used to collect data, conducted by two trained clinical psychologists. Individual structured interviews were used to collect data, conducted by two trained clinical psychologists. Qualitative information was collected by audio recording and transcribed texts from interviews with single participants. We used NVivo 11, a Computer Aided Qualitative Data Analysis Software (CAQDAS) program to manage the data.

## 3. Results

As described by Braun and Clarke [37], the approach used was a thematic analysis, described as a method for identifying, analyzing, and reporting models (themes) within the data. Based on the six-step guide suggested by Braun and Clarke, we first became familiar with the data by reading and re-reading the interview transcripts. Subsequently, we grouped the “recurring codes” for each theme to draw the test results. In thematic analysis, a theme is defined as a model found in the information that at least describes and organizes possible observations and interprets all the aspects of the phenomenon.

### Themes

The participants’ point of view was then summarized in six themes and related codes (Table 3).

1. Theme 1: Opinions related to smoking.

The issue of smoking is very delicate. All the interviewees declared a high concern for the health of smoking patients and showed a desire to help them quit. They also reported on problems related not only to health but to the conduct of daily life of these patients. In addition to the states of fatigue due to excessive consumption of cigarettes, the latter show evident signs due to smoking such as yellow teeth, clothes with holes, and a bad smell.

2. Theme 2: What is a cigarette for a schizophrenic patient?

All the answers led to a single conclusion: the cigarette for a patient suffering from schizophrenic spectrum disorders is the center of life. Everything revolves around cigarettes and the days are marked by these. Cigarettes, therefore, represent sharing, participation, and involvement with the group, and this causes addiction to increase every day. The double cigarette–coffee addiction was also highlighted, which is essential and of great emotional value for these patients.

3. Theme 3: Perception of a correlation between disorder and addiction.

The third question concerns the different adherence to smoking, concerning the severity of the disorder: in particular, this question was the precursor of an under-question that aims to identify periods in which patients need most to satisfy their addiction to cigarettes. The answers were very homogeneous as the operators of both A.T.C. reported that patients smoke a lot of cigarettes. However, based on what emerged from the interviews, there is no correlation between the disease and addiction. Therefore, no evidence can confirm the hypothesis of greater or lesser dependence on cigarettes for the severity of the disorder. However, the fact remains that most psychiatric patients are much more addicted to cigarettes than the rest of the population. All psychiatric patients smoke; they smoke a lot, and their addiction seems to be independent of the severity of the disorder. This theme leads to a second food for thought which refers to the periods when patients show a greater need for cigarettes.

4. Theme 4: When the addiction is greater.

The interviewees highlighted “critical” periods in which patients show greater addiction to cigarettes. In particular, periods of high stress linked, for example, to changes in drug therapy; seasonal changes or problems with family members or the therapeutic community cause patients to consume more cigarettes than usual.

5. Theme 5: Opinions on e-cigarettes.

E-cigarettes are well received by almost the entire sample. They are judged as “excellent substitutes for industrial cigarettes”. In total, 80% of the sample showed curiosity towards this new type of cigarette and even the remaining 20%, while still perplexed about the safety of e-cigarettes, still have a better opinion of them than commercial cigarettes.

6. Theme 6: How do the operators help patients?

This question made it possible to understand what behaviors are used by operators to help patients reduce or stop the use of cigarettes. In particular, it emerged that most of the sample, in addition to talking about the benefits that would be obtained by quitting smoking, implement strategies that tend to make people think less about cigarettes. These strategies involve activities that engage patients and keep them engaged so that they focus on the task at hand; this should ensure that the patients involved in these recreational moments do not focus their thoughts on cigarettes.

## 4. Discussion

This qualitative research has made it possible to better understand the motivations that push patients to use tobacco, the repercussions on a patient’s life, and the physical and mental health issues that smoking causes. In addition, a survey was carried out on the usefulness of e-cigarettes as a substitute for traditional cigarettes. Regarding the perception of smoking, the desire to help patients quit smoking or, at least, support them in an anti-smoking process, was underlined by the interviewees. The alternatives to smoking are numerous; among the intervention programs and the possible solutions, there are transdermal patches that release nicotine or nicotine chewing gum that reduce cravings. Furthermore, the danger of smoking was highlighted [38]. All the participants in the study reported that a cigarette is a product that hurts a lot and leads to numerous negative implications such as yellow fingers and a bad smell in the environment and on clothes. In addition, cigarette butts often burn patients’ clothes, linens, curtains, and sofas. There are many problems encountered on a physical level: the operators reported that patients have no physical resistance, are very tired during the day, and often report annoyances related to the use of cigarettes such as nausea and headaches. Finally, the operators reported that patients often do not understand that excessive consumption of cigarettes seriously harms their health and is a major cause of death. The value that cigarettes have for psychiatric patient is very strong. The cigarette represents the daily life of the individual, who marks his/her days. There are many moments in which cigarettes are used by patients: cigarettes are seen as a moment of sharing and socializing with other patients and are also used as a means of communication between them [39]. They represent the emblem of their personality. Many aspects suggest the rituality attributed to cigarettes. The operators highlight the different types of behaviors towards these: some use cigarettes to calm down, others prefer to use tobacco to ‘create them’ for themselves; others prefer to smoke with others and some smoke on the sidelines. In all cases, the cigarette represents the center of their actions; the final and initial cause of the behavior of these patients. Cigarettes have an adaptive value to society: they help patients to feel less alone, to be part of a group in which they share moments and thoughts, and are often idealized as an anti-stress tool. The high consumption of cigarettes has numerous implications, but patients, especially the most addicted, do not deprive themselves of smoking even when they feel at risk or become sick and have to stay in bed, as the benefit they receive is greater for them than the harm carried out to one’s health [40,41]. From the interviews released by the operators, no correlation between cigarette addiction and the severity of the disorder was highlighted. However, it was highlighted that most psychiatric patients show a high dependence on cigarette smoking and periods in which patients show a higher need for nicotine were highlighted. These are periods of high stress, which lead patients to smoke more cigarettes. Among the reasons that lead to high levels of stress we find seasonal change: especially with the onset of colder periods, patients show a stronger dependence and tend to ask operators for more cigarettes than usual; change in drug therapy: between one change in therapy and another, and therefore the decompensation brought about by therapies, especially at the beginning, patients are much more prone to smoking cigarettes; practitioners believe that patients perceive cigarettes as something that can counteract psychiatric drugs: they have a relaxing effect, while cigarettes lead to over-excitation in patients. Stress is linked to moods: many reasons could change the mood of patients that are already prone to emotional instability such as family problems, discomfort between companions, and periods of feeling down, which are all factors that lead patients to consume a higher number of cigarettes. Among non-smoking operators, the opinion is unanimous: it is a product that is harmful to health but which is still much less risky than industrial cigarettes. Smoking operators also reported a very positive opinion regarding electronic cigarettes. Some see them as valid substitutes for industrial cigarettes and some argue that they are two different things; in both cases, the opinion on the effectiveness in decreasing the use of cigarettes remains unchanged: they all agree that electronic cigarettes are a good alternative to cigarettes. Among the factors that increase the positive vision of e-cigarettes are: they do not release bad smells; they reduce the problems related to resistance; they do not stain or burn clothes and linen. The operators are confident in the research and hope that over the years, e-cigarettes will be used much more than industrial cigarettes.

## 5. Conclusions

This research has deepened the point of view of the health personnel who accompany patients every day in their psychosocial rehabilitation paths. From the responses of the operators, it emerged that their opinion on smoking, regardless of whether they are smokers or not, is very negative. They also reported all the same signs of annoyance towards traditional cigarettes, such as bad smells, nausea, or yellow hands and teeth. As far as the patients are concerned, it was shown that they are almost all smokers but that their addiction is not caused by the severity of the disease. It has not emerged that patients with more serious ailments can have greater adherence to the use of cigarettes. Periods of high stress due to various factors such as seasonal change, change of therapy, health problems, family problems, and general concerns were reported to lead patients to consume a higher number of cigarettes. As for the perception concerning low-risk products such as electronic cigarettes, almost all respondents reported consent to their use, some of them are concerned about the long-term outcomes due to the prolonged use of this new type of cigarette but they are still convinced that they do less harm than industrial cigarettes. E-cigarettes were also defined as viable substitutes for cigarettes and the majority of smoking respondents either use them or have used them to quit smoking. Finally, to decrease the use of cigarettes, health professionals have used various methodologies. In addition to being warned by staff about the risks of smoking, they are engaged in activities that require active participation. Among the activities that are carried out most frequently, we find, for example, painting or hiking. In this way, patients shift their attention from cigarettes to the task and actual improvements were found in patients.

## Figures and Tables

**Table 1:** Participants’ characteristics.

Socio-Demographic Characteristics	*n* (%)
Gender	
Male	9 (45)
Female	12 (55)
Age	
Age range	
20–30	3 (15)
30–40	6 (30)
40–50	10 (50)
50–60	1 (5)
Role	
Psychiatrist	3 (15)
Psychologist	8 (40)
Pedagogist	1 (5)
Social Worker	1 (5)
Nurse	5 (25)
Social Health Operator	2 (10)

**Table 2:** Research questions.

Interview
1. “What is your opinion on cigarette smoking?”
2. “What does the cigarette represent for a patient with schizophrenia?”
3. “Concerning the severity of the disorder, do the patients show a greater/lesser dependence on cigarettes?”
4. “What do you think of e-cigs?”
5. “What do you do to help your patients with this?”

**Table 3:** Themes and Codes.

	Themes	Codes
1	Opinions about smoking	• Perception of the negative consequences of smoking • Patient health
2	What is the cigarette for people with schizophrenia?	• Reasons to smoke • The ritual aspect of the cigarette
3	Perception about a correlation between disorder and addiction	• The severity of the disorder • Dependence
4	When the addiction is greater?	• Periods of stress • Seasonal change • Change in therapy
5	Opinions on e-cigarettes	• E-cigarettes as a substitute for the cigarette • Perplexity
6	Strategies for helping patients	• Recreational activities to distract yourself • Educate patients about the risks of smoking

## Data Availability

Not applicable.
